# Surgical Treatment for the Ankle and Foot in Patients with Rheumatoid Arthritis: A Systematic Review

**DOI:** 10.3390/jcm9010042

**Published:** 2019-12-24

**Authors:** Ana Belen Ortega-Avila, Antonio Moreno-Velasco, Pablo Cervera-Garvi, Magdalena Martinez-Rico, Esther Chicharro-Luna, Gabriel Gijon-Noqueron

**Affiliations:** 1 Department of Nursing and Podiatry, Faculty of Health Sciences, University of Malaga,29071 Malaga, Spain; amv150_antonio@hotmail.com (A.M.-V.); pcervera@uma.es (P.C.-G.); magdalenamr96@gmail.com (M.M.-R.); gagijon@uma.es (G.G.-N.); 2 Department of Behavioural and Health Sciences, Nursing Area, Faculty of Medicine, Miguel Hernández University, San Juan de Alicante, 03550 Alicante, Spain; ec.luna@goumh.umh.es; 3 Biomedical Research Institute (IBIMA), 29010 Malaga, Spain

**Keywords:** surgical treatment, rheumatoid arthritis, foot, ankle, systematic review

## Abstract

The aim was to identify effective surgical treatments for patients with rheumatoid arthritis in the foot and/or ankle. A systematic review of the literature was conducted via a data search of the PubMed, Scopus, Cumulative Index to Nursing and Allied Health Literature (CINAHL), and PROSPERO databases, from their inception until June 2019. Only non-randomized controlled trials and cohort studies were included in this review. Two of the present authors independently assessed the quality of each study and extracted the relevant data. A quality assessment of all articles was performed using the methodological index for non-randomized studies criteria. In addition, the Newcastle Ottawa scale was used for cohort studies. Thirteen studies met the inclusion criteria (five cohort studies and eight NRCTs). The total population considered was 923 patients (570 patients had RA), with a mean age of 58.8 years. Regarding the risk of bias, both the NRCTs and the cohort studies had a moderate level of quality. Despite the relatively low quality of these studies, surgical treatment for the foot and/or ankle is shown to reduce pain and improve functionality in patients with RA, in the short term (6–12 months).

## 1. Introduction

Rheumatoid arthritis (RA) is a systemic, chronic, autoimmune, and polyarticular pathology that produces inflammation, which mainly affects the synovial joints, with progressive cartilage degeneration and erosion, leading to pain and functional limitation [1,2,3,4].

The aetiology of RA is unknown, but there are predisposing risk factors, some of which may be genetic, such as the presence of the Human Leucocyte Antigen (HLA)-DR4 class II histocompatibility antigen. Other relevant factors are gender, women are three times more likely than men to have RA [5]. The prevalence of RA is high, at 0.3%–1.2% and it increases with age (6% higher among those aged 65 years or more) [6].

RA most commonly affects the small joints in the upper limbs, in the hand and the wrist [6], and in the lower ones, in the ankle and foot (90%). The greatest number of deformities are found in the forefoot [7]. Foot problems are strongly related to RA and often develop rapidly; thus, half of all RA patients present foot problems within three years of diagnosis [8]. RA often takes the form of symmetrical small joint polyarthritis, with pain, swelling, and/or stiffness. The symptoms are usually first observed in the forefoot [9] and include hallux valgus, subluxation, and dislocation or erosion of the metatarsophalangeal (MTP) joints, together with hammer toes or claw toes in the lesser toes [10]. RA can also affect the subtalar and mid-tarsal joints and in some cases the ankle joint. In the subtalar joint, synovitis causes pain and stiffness leading to cartilage loss and bone erosion. These changes increase the valgus deformity, causing flattening of the longitudinal arch. Synovitis of the MTP-joint is related to forefoot deformity [11,12].

Clinical practice offers many conservative treatments to relieve the pain of RA, including non-steroidal anti-inflammatory drugs, standard disease modifying anti-rheumatic drugs, biological treatments, specialised footwear and orthotics. [13,14]. If these approaches do not satisfactorily resolve the pain, deformity, or loss of function, or facilitate the use of conventional shoes, various types of surgical treatment may be proposed [15], regardless of the stage of the disease, to be used initially as prophylaxis, and at more advanced levels to correct deformities, recover foot function, and relieve pain [12,16]. Surgical techniques are most commonly applied to the forefoot [17], but the hindfoot and ankle are also treated [18]. The surgical procedures for the forefoot are arthrodesis of the first MTP joint and/or arthroplasties of the MTP joints [19,20] and surgical treatments to the ankle are total arthroplasty and prosthesis [21,22]. Studies have been conducted to describe and examine the role played by different surgical techniques for the foot and ankle for patients with RA [23,24,25] but to date no systematic review has been undertaken to assess their impact on pain relief and improved functionality.

Accordingly, the present paper aims mainly to identify effective surgical treatments for patients with RA in the foot and/or ankle.

## 2. Methods

The review protocol is registered at the International Prospective Register of Systematic Reviews (PROSPERO).

### 2.1. Design

This review was performed in accordance with the Preferred Reporting Items for Systematic Reviews and Meta-Analyses (PRISMA) statement [26].

### 2.2. Search Strategy

One member of the research team (ABOA) carried out the literature search, after establishing its aims and checking that no previous studies in this respect had been carried out. The PROSPERO, PubMed, Scopus, and Cumulative Index to Nursing and Allied Health Literature (CINAHL) databases were searched, from their inception until June 2019, using the optimized search strategies described in Appendix A.

### 2.3. Eligibility Criteria

The PICO (P = population; I = intervention; C = comparator; O = outcome) and the PECO (P = population; E = exposition; C = comparator; O = outcome) frameworks were applied by two reviewers (PCG-GGN), to implement the following inclusion criteria for the studies considered.

- Study population composed of adults aged at least 18 years, diagnosed with RA, and who underwent surgery to the ankle and/or foot.
- Study design: Randomised controlled clinical trials (RCTs) and non-randomised controlled clinical trials (NRCTs) in which surgical treatment to the ankle or foot was performed for patients with RA who experienced pain and were disabled were assessed during the follow-up period
- Cohort studies of patients with RA and/or without RA to evaluate one or more surgical treatments to the foot and/or ankle, with a follow-up period.
- Studies which assessed pain and disability by the following outcomes: Foot Function Index [27], American Orthopaedic Foot and Ankle Score [28], Japanese Society of Surgery of the Foot ankle/hindfoot scale [29,30], Ankle Osteoarthritis Scale [31], Kofoed ankle score, Salford Rheumatoid Arthritis Foot Evaluation- Questionnaire [32], American College of Foot and Ankle Surgeons scoring scale [29], Short-Form 36 Questionnaire [33], or Stanford Health Assessment Questionnaire disability index [34]

The following types of studies were excluded from consideration:

- Studies that examined surgical treatments to the foot or ankle in individual patients with RA (case studies).
- Studies combining conservative and surgical treatment to the foot or ankle in patients with RA, including those describing a relevant post-operative rehabilitation programme.
- Studies assessing pain or disability in juvenile arthritis.
- Studies where the first treatment options were alternatives such as orthopaedic shoes, foot or physiotherapy, not surgery.

### 2.4. Study Selection

Two blinded reviewers (A.M.-V and M.M.-R) evaluated the search results. The reference lists were reviewed independently to observe the fulfilment or otherwise of the inclusion criteria. Disagreements were resolved by discussion between the two reviewers, and if consensus was not possible, further opinion was sought (from A.B.O.-A, P.C.-G, E.C.-L and G.G.-N). We also planned, if necessary, to send an email to the original authors to obtain further information regarding the study findings, but this measure was not needed for any study.

### 2.5. Data Extraction

The titles and abstracts were then reviewed independently by two reviewers (AMV and MMR) and full-text versions of the articles considered relevant were obtained. The same reviewers performed the second stage of screening, reading the full text of the selected articles. The following data were extracted from each study, using a standardized template: study details (author and year of publication), study participant characteristics (mean age, sex, details of the foot affected), study design (cohort study or NRCT), follow-up time, and measurement instrument used (mean and SD).

No meta-analysis was carried out, due to the heterogeneity of these studies and the varying outcomes described.

### 2.6. Quality Assessment of the Studies Included

Two researchers independently assessed the risk of bias in the articles included, using the Methodological Index for Non-Randomised Studies (MINORS) for the NRCTs [35] and the Newcastle-Ottawa Scale (NOS) [36] for the cohort studies.

The MINORS is used to assess the internal and external validity of non-randomised surgical studies (comparative and/or non-comparative). It consists of twelve items, of which eight are used to assess both comparative and non-comparative studies and four are exclusive to comparative studies. The items are scored on the following scale: 0 (not reported), 1 (reported but inadequate) or 2 (reported and adequate). The overall maximum score is 16 for non-comparative studies and 24 for comparative ones. For non-comparative studies, the following range of scores is applied: 0–4, very low quality; 5–8, low quality; 9–12, moderate quality; and 13–16, high quality. For comparative studies, the corresponding scores are 0–6, very low quality; 7–12, low quality; 13–18, moderate quality; and 19–24, high quality [35].

The NOS is a reliable, valid tool for assessing the quality of cohort studies. In this case, the quality score is based on three categories: group selection (four items), inter-group comparability (one item), and outcome and exposure assessment (3 items). A maximum of one point is awarded for each item in the group selection and in the outcome and exposure assessment categories. A maximum of two points is awarded for comparability. Thus, the maximum score with the NOS is 9 points, which represents the highest methodological quality [36].

## 3. Results

**Figure 1:**
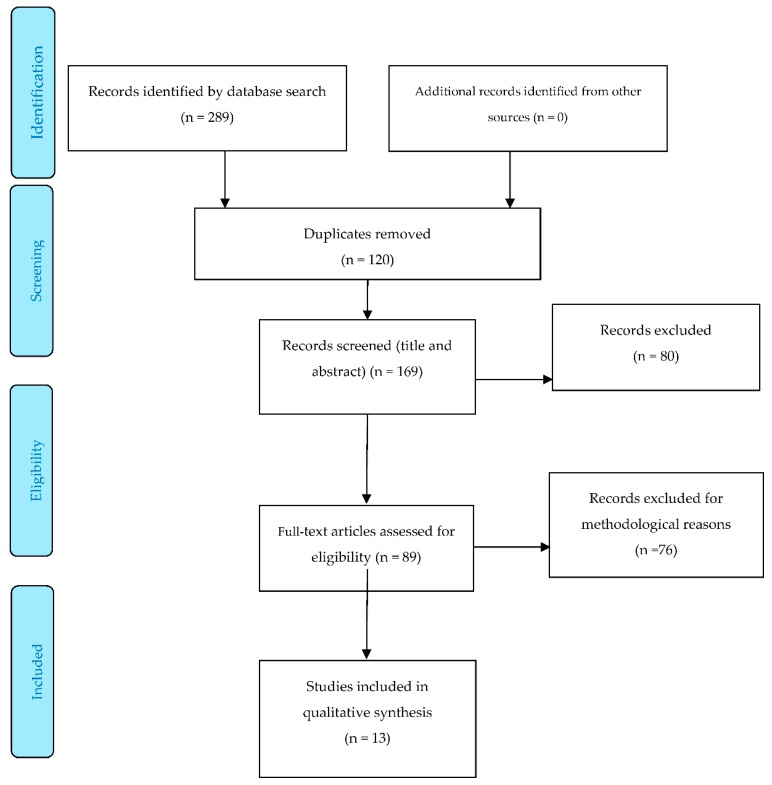
PRISMA Flow Diagram.

Initially, 289 studies were identified, but 120 were duplicated among the different databases. The remaining 169 were screened against our inclusion/exclusion criteria, using the titles, abstracts, and key words. Eighty-nine studies met the inclusion criteria. After quality appraisal, a further 76 were excluded, and so 13 studies remained in the final analysis. Five of these were cohort studies and eight were NRCTs. None were RCTs. Figure 1 shows the PRISMA flow diagram for the studies included in the review [37].

### 3.1. Study Characteristics

The 13 studies included a total of 923 patients, with a mean age of 58.8 years. Of these, 570 (90.7% of whom were female) had RA. Surgical treatment was predominantly performed on the right foot or ankle (*n* = 173), although in many of the studies the location was not specified.

The NRCTs included a total of 548 patients (82.48%were women), with a mean age of 58.46 years. The minimum follow-up period was twelve months (mean duration: 44.75 months). The most frequent location of the surgical treatment was in the metatarsophalangeal (MTP) joints and the main technique used was arthroplasty (Table 1).

The cohort studies included a total of 393 patients (75.31%were women), with a mean age of 59.34 years. The minimum follow-up period was six months (mean duration: 39.8 months). The most frequent location of the surgical treatment was in the first MTP joint, or in various MTP joints, by arthrodesis or arthroplasty (Table 2).

All studies reported a considerable improvement in pain relief as well as in functional capacity after surgery in the patients with RA, regardless of the technique used (arthroplasty or osteotomy or arthrodesis) or the location of the surgery (MTP joints or small toes or ankle). This combination of techniques achieved the best results, both in relieving pain and in enhancing functionality (*p* < 0.001). Table 3 summarized the data that help assess the actual outcome of each of the interventions in the studies that were included in this review.

### 3.2. Risk of Bias

None of the NRCTs included achieved the maximum MINORS score. The best comparative study received an overall MINORS score of 20 out of 24, and the mean overall score for all comparative studies was 18. The areas of weakest reporting for the comparative studies were the unbiased assessment of the study endpoint, the inclusion of consecutive patients and the use of endpoints appropriate to the study aim. The prospective recruitment of patients and the use of appropriate statistical analyses scored more highly. The best non-comparative study received an overall score of 11 out of 16, and the mean overall MINORS score for all non-comparative studies was 10. The areas of weakest reporting for the non-comparative studies were the unbiased assessment of the study endpoint, the prospective calculation of the study size, the inclusion of consecutive patients, the use of endpoints appropriate to the study aim, and the use of a follow-up period appropriate to the study aim. Table 4 shows the MINORS score obtained by each study.

In the cohort studies, the scores obtained ranged from five to seven points (out of a possible nine). Two out of four cohort studies scored seven points. All the studies allocated sufficient time for follow-up. The critical appraisal details are presented in Table 5.

## 4. Discussion

This review has two main aims: to identify the surgical treatment provided to the foot and ankle for patients with RA and to evaluate the risk of bias in studies conducted in this field in order to assess their quality.

Regarding the main objective, many studies have examined records of surgical treatment for the foot and ankle in patients with RA, concluding that the outcome is often very beneficial [41,42,43]. Deformities at this level can play an important role in determining the pain experienced and in provoking loss of functionality. Moreover, they directly reduce the patient’s ability to perform the activities of daily life and may even influence mood [42].

Our findings are based on NRCTs and cohort studies, which show that surgical treatment for these patients usually decreases pain and enhances functionality, assessed over a follow-up period of at least 12 and six months, respectively [19,25]. All of these studies used an objective measuring instrument, providing validity, reliability, and responsiveness, underpinning the measurements obtained for the clinical variables observed in the patients after the surgical technique in question had been performed [25]. These instruments provide a clinical assessment of both pain and functionality, and were used to indicate the change achieved (pre/post-surgery). Despite the objective results obtained for the clinical variables, as regards pain and functionality, we were unable to perform a meta-analysis because the same instrument was not used in every study considered and so the data reported are heterogeneous. In addition, the follow-up time was not uniform, and this factor may have influenced the findings reported, as longer follow-up times after a surgical intervention take into account a greater potential number of recurrences or post-operative complications, thus enabling a more reliable analysis [34,44].

For patients with RA studies, the most common surgical procedures investigated are arthrodesis of the first MTP joint and/or arthroplasties of the MTP joints [19,20,38,43], since these patients are particularly subject to deformities in the forefoot (such as hallux valgus, hallux rigidus, floating of the lesser toes or synovial inflammation of the MTP joints) or in the hindfoot (such as hindfoot valgus or flat foot), which can aggravate pain and disability among this population [10,17]. This combination of techniques was most commonly used in the studies considered and achieved the best results, both in relieving pain and in enhancing functionality (*p* < 0.001) [19,20,40,43]. However, there is no evidence that this is more effective than arthroplasty in all the metatarsal heads (or the fist metatarsal), because according to the papers included in our analysis, the only related research conducted were case studies [18,45,46], which are considered to present low methodological quality. Moreover, these papers do not address the specific object of our investigation. In contrast to the arthroplasty of the first MTP joint, there is a tendency to preserve the joint by performing shortened osteotomies in the diaphysis of the metatarsals. According to Horita et al. [38] and Ebina et al. [39], the reduction in pain level in the osteotomy group differs from that achieved by arthroplasty, although in terms of functionality, the results are very similar in both cases. Regarding surgical treatments to the ankle, the only studies identified were those by Su et al. [21] Van der Heide et al. [24] and Pedersen et al. [22]. In these cases, the most commonly investigated surgical procedure was that of total arthroplasty, followed by the use of a prosthesis. All these investigations reported improved functionality and reduced pain, although the differences between groups were not statistically significant.

Regarding risk of bias, the results obtained show that the majority of NRCTs considered, both comparative and non-comparative, presented only moderate quality. Only one comparative study was rated as ‘high quality’, with 20 points. The weaknesses common to all the NRCTs were the non-inclusion of consecutive patients, the failure to define endpoints appropriate to the study aim, and the presence of bias in assessments made at the study endpoint. It is important to highlight these deficiencies in the studies considered, since these shortcomings may have significant repercussions in the context of scientific research. If patients are recruited during the investigation, or if large numbers of participants drop out during the course of the study, the results obtained may not be valid or reliable. Furthermore, an unambiguous explanation should be given of the criteria used to assess the main outcome of the study, and this should always be in accordance with the research question originally posed. If the study aim is to compare techniques or population groups, there must be a blind evaluation of the outcomes achieved and a double-blind evaluation of subjective endpoints to provide trustworthy results as a basis for future investigation, and to demonstrate the suitability or otherwise of the research method applied. With respect to the possible presence of bias, the cohort studies considered presented only moderate methodological quality. All were deficient in the selection of the non-exposed cohort and assessment of outcomes. Both of these questions should be taken into account in research of this type, since it is difficult to identify the type of population being evaluated if there is no clear description of the non-exposed cohort and the main outcome cannot be properly assessed in the absence of a blinded independent evaluator.

Recent investigations indicate that patients with RA in the foot and/or ankle commonly receive surgical treatment, which achieves beneficial effects regarding pain relief and enhanced functionality [8,15]. With regard to the specific area of investigation, most of the studies considered focused on the treatment of the forefoot. However, few studies in this area present sufficient methodological quality, the majority being mere series of case studies. Moreover, the size of the sample used, and the follow-up period of the patients involved are in many cases inadequate.

The present systematic review presents numerous strengths: to our knowledge, this is the first such review focusing on surgical treatment to the foot and ankle for patients with RA in which specific review tools are applied to analyse the risk of bias, the study population and the application of a rigorous methodological process. Moreover, our review is based on a literature search of five medical databases, with no limitation on time. Nevertheless, it is also subject to certain limitations. The first is the small number of NRCTs and cohort studies included. In addition, the data considered present considerable heterogeneity, making it impossible to carry out a meta-analysis and hampering the overall assessment made. Finally, the studies reviewed presented a relatively high risk of common bias.

Our findings highlight the problems encountered in clinical practice and in research studies to determine whether a given surgical technique is appropriate for a patient with RA affecting the foot, and the need for further investigation to improve this process. Most of the studies considered applied objective instruments of pain and functionality, in the view that these questions of RA not only influence the activities of daily life, but also have an affective and psychological impact. However, there is no unified criterion as to the most suitable instrument for this purpose, the best follow-up time after surgery (periods of 12–76 months have been cited), or the number of patients to be included in the study population. Attention should be paid to possible confounders, such as adverse treatment effects, recurrences, treatment modality, current pattern of medication, and the duration of the disease. All of these questions are relevant and appropriate consideration of their effects might obviate the need for multiple reinterventions. Surgical interventions are conducted to achieve proper alignment, but also to ensure the functionality of the joint in the foot and/or ankle, and thus prevent recurrence [44].

## 5. Conclusions

Despite the above-stated limitations, the study findings clearly show that surgical treatment to the foot and/or ankle is beneficial as regards reducing pain and improving functionality in patients with RA (6–12 months). However, the deficient quality of many of the studies considered limits the extent to which definitive conclusions can be drawn. Future research of this type is needed, ensuring homogeneity in the study groups by size and composition, as well as in aspects such as the follow-up period considered and the description of main outcomes, in order to minimise the risk of bias.

## Figures and Tables

**Table 1:** Characteristics of Non-Randomized Controlled Trials (NRCTs).

Author Year	Patients (n)	Age (Years)	Sex		Foot/Ankle			Follow up	Surgical Site	Surgical Technique	Outcome
			Female	Male	Left	Right	Bilat				
Horita et al., 2018 [38]	29 RA	64.2 ± 22	29	0	34 feet		5	34.75 months (min. 24 months)	MTP joints	Arthroplasty Resection group (*n* = 14)	JSSF
										Osteotomy Joint preservation group (*n* = 15)	
Ebina et al., 2017 [39]	49 RA	63.45	46	3	63 feet		14	45 months	MTP joints	Arthroplasty or osteotomy Resection-replacement (*n* = 28) Preserving (*n* = 35)	SAFE-Q
Fukushi et al., 2016 [40]	17 RA	62	16	1	12	11	5	68 months	MTP joints	Arthrodesis of the 1st MTP joint and arthroplasty of the remaining target areas. Resection arthroplasty (RA) (*n* = 13)	JSSF, Hallux and lesser toes scale
										Osteotomy of all the toes Joint preservation (*n* = 10)	
Bhavikatti et al., 2012 [41]	49 RA	56.1 ± 26	44	5	66 feet		17	51 months (40–65)	MTP joints	Osteotomy	AOFAS
Benoni et al., 2012 [42]	258 (193 RA)	64 ± 33	212	65	162 feet and 31 ankles		-	12 months	Ankle and foot	Arthrodesis or osteotomy of the forefoot. Stainsby ankle group (*n* = 31)	SF-36 HAQ
										Arthrodesis of the hindfoot. Arthrodesis or arthroplasty of the ankle. Feet group (*n* = 162)	
Rosenbaum et al., 2011 [19]	53 RA	53 ± 9	43	10	58 feet		5	41 ± 12 months	MTP joints	Arthrodesis of the 1st MTP joint. Arthrodesis (*n* = 33)	HAQ FFI
										Arthroplasty of all target areas. Arthroplasty (*n* = 25)	
Van der Heide et al., 2009 [24]	58 (54 RA)	55 ± 27	48	10	58 ankles		4	31 months	Ankle	Total arthroplasty of the ankle STAR (*n* = 37) BP (*n* = 21)	Kofoed score
Su et al., 2004 [21]	17 RA	50 ± 31	14	2	27 ankles		10	76 months	Ankle	Total arthroplasty of the ankle HSS Custom (*n* = 7) Endotec (*n* = 19)	AOFAS

RA: Rheumatoid Arthritis; MTP: Metatarsophalangeal; JSSF: Japanese Society of Surgery of the Foot; SAFE-Q: Salford Rheumatoid Arthritis Foot Evaluation- Questionnaire; AOFAS: The American Orthopaedic Foot and Ankle Score; HAQ: Health Assessment Questionnaire; SF-36: Short Form-36; FFI: Foot Function Index.

**Table 2:** Characteristics of cohort studies.

Author Year	Patients (n)	Age (Years)	Sex		Foot/Ankle			Follow up	Surgical Site	Surgical Technique	Outcome
			Female	Male	Left	Right	Bilat				
Fazal et al., 2018 [20]	26 (10RA)	64 ± 15	23	3	32 feet		6	49 months (40–62 months)	1st MTP joint	Arthrodesis: fixation with 2 orthogonal plates	AOFAS FADI
Donegan et al., 2017 [43]	228 (29RA)	55.8 ± 3.2	178	50	122	140	22	7.6 ± 1.64 months	1st MTP joint, small toes.	Arthrodesis of the 1st MTP joint with double screw fixation and arthroplasty of the small toes	ACFAS scoring scale and SF-36
Pedersen et al., 2014 [22]	100 (50RA)	60	64 (35 RA)	36 (15 RA)	58 RA ankles 54 NRA ankles		12	64.7 ± 22.1	Ankle	Total arthroplasty of the ankle	AOS SF-36
Dodd et al., 2011 [25]	16 (4RA)	56 ± 21	12 (3 RA)	3 (1RA)	24 (6 RA)			14 months (min. 6 months)	Small toes	Standby	Manchester Oxford Foot and Ankle Score
Thomas et al., 2006 [44]	23RA	60.9 ± 22	19	4	21	22	20	64.9 months (22–108)	MTP joints	Arthroplasty	AOFAS VAS

RA: Rheumatoid arthritis; NRA No-rheumatoid arthritis; MTP: Metatarsophalangeal; AOFAS: The American Orthopaedic Foot and Ankle Score; FADI: Foot and Ankle Disability Index; ACFAS: The American College of Foot and Ankle Surgeons; SF-36: Short-Form 36; AOS Ankle Osteoarthritis Scale; VAS: Visual Analogue Scale.

**Table 3:** Outcomes in pain relief and functional capacity in included studies.

Author	Surgical Site	Outcome	Surgical Technique							
Horita et al. [38]	MTP Joints		Arthroplasty resection group				Osteotomy joint-preservation group			
		JSSF (0–100 points)	Pre		Post	*p-*Value	Pre	Post	*p-*Value	
		Pain	22.5 ± 10		28.1 ± 10	0.003	20.0 ± 20	28.9 ± 10	0.001	
		Deformity	12.2 ± 12		19.9 ± 13	0.001	14.7 ± 6	22.0 ± 6	0.001	
		Range of motion	10.1 ± 15		12.0 ± 15	0.023	10.9 ± 7	14.3 ± 2	0.004	
		Walking ability	13..1 ± 10		18.8 ± 10	0.003	11.4 ± 7	18.9 ±	0.003	
		Activities of daily life (ADL)	3.3 ± 8		5.4 ± 8	0.015	5.2 ± 3	7.1 ± 2	0.012	
		JSSF-RA scale (total)	61.3 ± 19		83.9 ± 27	0.001	62.2 ± 15	90.8 ± 8	0.001	
Ebina et al. [39]	MTP Joints		Resection-replacement group				Preserving group			
		SAFE-Q (0–100 points)	Pre		Post	Change	Pre	Post	Change	
		Pain	36.8		75.0	38.2	42.2	82.6	44.4	
		Physical functioning and daily living	43.2		68.8	25.6	52.7	78.1	25.4	
		Social functioning	44.3		72.0	27.7	52.5	81.9	29.4	
		General health and well-being	48.4		68.4	20.0	45.5	84.4	38.9	
		Shoe-related	30.1		50.3	20.2	30.6	64.4	33.8	
Fukushi et al. [40]	MTP Joints		Joint-preserving			Resection arthroplasty			*p-*Value	
		JSSF (0–100 points)	Pre		Post	Pre	Post		Pre	Post
		Hallux	Total	61.4 ± 16.3	88.2 ± 8.1	54.5 ± 8.3	79.4 ± 9.6		0.196	0.035
			Pain	27.0 ± 11.6	37.0 ± 4.8	26.9 ± 4.8	36.4 ± 6.7		0.982	0.808
			Function	32.8 ± 5.3	36.2 ± 6.0	27.5 ± 5.5	28.0 ± 3.6		0.032	0.001
			Alignment	3.1 ± 5.3	15.0 ± 0.0	0.0 ± 0.0	15.0 ± 0.0		0.047	-
		Lesser toes	Total	57.4 ± 19.2	87.7 ± 8.3	45.2 ± 12.3	73.6 ± 13.9		0.091	0.015
			Pain	24.4 ± 7.3	36.6 ± 5.0	23.8 ± 2.3	36.4 ± 5.0		0.867	0.894
			Function	28.2 ± 11.3	36.1 ± 6.8	21.4 ± 2.4	30.3 ± 5.7		0.077	0.050
			Alignment	3.5 ± 4.2	15 ± 0.0	0.0 ± 0.7	7.0 ± 6.3		0.006	0.001
Bhavikatti et al. [41]	MTP Joints		Osteotomy group							
			Pre			Post			Change	
		AOFAS (0–100 points)	39.8 (18–56)			88.7 (48–92)			48.9	
Benoni et al. [42]	Ankle and foot		Feet group				Stainsby ankle group			
			Pre		12 months	*p-*Value	Pre	12 months		*p-*Value
		HAQ (0–3points)	1.0		0.96	0.8	1.4	1.4		0.8
		SF-36 (0–100 points)								
		Physical functioning (PF)	44		51	<0.001	27	33		0.2
		Social functioning (SF)	73		75	0.4	-	-		-
		Role limitations because of physical health problems (RP)	34		42	0.03	19	27		0.4
		Bodily pain (BP)	40		48	<0.001	34	46		0.03
		General mental health (GH)	51		49	0.4	51	46		0.2
		Emotional problems (RE)	64		67	0.5	51	55		0.7
		Vitality (VT)	51		50	0.5	38	45		0.2
Rosenbaum et al. [19]	MTP joints		Arthrodesis				Arthroplasty		*p-*Value	
		HAQ (0–3)	1.0 ± 0.7				0.9 ± 0.6		Ns	
		FFI (0–100)	32 ± 19				30 ± 17		Ns	
van der Heide et al. [24]	Ankle						STAR		BP	
							Follow up (31 months)			
		Kofoed Score (0–100 points)	Mean Kofoed score73 points (SD16, range 21–92)		<50 points		4		0	
					Removal of implant		4		1	
					>50 points		29		20	
Su et al. [21]	Ankle		HSS Custom				Endotec		*p*-value	
		AOFAS (0–100 points)	85				79		0.12	
Fazal et al. [20]	1st MTP joint		Arthrodesis group							
			Pre		Post				Change	
		AOFAS (0–100 points)	37.1 ± 8.8		80.7 ± 8.8				43.6 (*p* < 0.0001)	
		FADI (0–100 points)	40.3 ± 11.0		86.9 ± 14.2				46.6 (*p* < 0.0001)	
Donegan et al. [43]	1^st^ MTP joint				Arthrodesis of the 1st MTP joint with double screw fixation and arthroplasty of the small toes					
					Post					
		SF-36 (0–100 points)	Physical function		79.5 ± 2.6					
			Energy		72.3 ± 1.7					
			Painlessness		79.4 ± 1.5					
			General health		73.0 ± 1.9					
		ACFAS (0–68 points)	Subjective score		37.2 ± 2.5					
			Objective score		14.5 ± 1.7					
Pedersen et al. [22]	Ankle				Arthroplasty group					
					Pre		Post		Change	
		AOS (0–100 points)	Pain		63.3 ± 16.6		18.5 ± 17.8		−44.8 ± 22.7	
			Disability		70.0 ± 14.6		30.0 ± 23.4		−40.0 ± 23.0	
		SF-36 (0–100 points)	Physical component		27.4 ± 8.0		34.4 ± 8.8		6.9 ± 8.6	
			Mental component		59.0 ± 13.4		63.1 ± 9.5		4.1 ± 12.8	
Dodd et al. [25]	Small toes				Standby					
					Pre		Post		Change	
		Manchester and Oxford Foot and Ankle Score	Pain (0–20)		13.75 ± 3		8.75 ± 3		−5	
			Social (0–16)		11.5 ± 10		7.5 ± 11		−4	
			Walking (0–28)		22.5 ± 8		14.75 ± 10		−7.75	
Thomas et al. [44]	MTP Joints				Arthroplasty group					
					Final follow-up					
		VAS (0–10 points)	Pain at rest		1.8 ± 6.1					
			Pain in motion		4.1 ± 8.9					
		AOFAS (0–100 points)			64.5 ± 27					

RA: Rheumatoid arthritis; MTP: Metatarsophalangeal; Pre: Pre-operation; Post: Post-operation; JSSF: Japanese Society of Surgery of the Foot; SAFE-Q: Salford Rheumatoid Arthritis Foot Evaluation-Questionnaire; AOFAS: The American Orthopaedic Foot and Ankle Score; HAQ: Health Assessment Questionnaire; SF-36: Short Form-36; FFI: Foot Function Index¸ ACFAS: The American College of Foot and Ankle Surgeons; AOS Ankle Osteoarthritis Scale; VAS: Visual Analogue Scale.

**Table 4:** Quality assessment of the NRCTs studies considered, according to the MINORS score.

Author	Clearly Stated Aim	Inclusion of Consecutive Patients	Prospective Collection of Data	Endpoints Appropriate to the Aim of the Study	Unbiased Assessment of the Study Endpoint:	Follow-up Period Appropriate to the Aim of the Study	Loss to Follow up Less than 5%	Prospective Calculation of the Study Size	An Adequate Control Group:	Contemporary Groups	Baseline Equivalence of Groups	Adequate Statistical Analyses	Score 24/24 Or 16/16
Horita et al. [38]	2	1	2	1	0	2	2	2	1	2	1	2	18/24
Ebina et al. [39]	2	1	2	2	0	2	2	2	1	2	2	2	20/24
Fukushi et al. [40]	2	1	1	2	0	0	2	2	2	2	2	2	18/24
Rosenbaum et al. [19]	2	1	1	1	0	2	2	2	2	2	1	2	18/24
Su et al. [21]	2	1	2	1	1	2	2	2	1	0	0	2	16/24
Bhavikatti et al. [41]	2	1	2	1	0	2	2	0					10/16
Benoni et al. [42]	2	2	2	2	0	1	0	2					11/16
van der Heide et al. [24]	2	1	1	1	0	1	2	1					9/16

Rating: 0 (not reported), 1 (reported but inadequate), 2 (reported and adequate). Overall maximum score: 16/16 for non-comparative studies and 24/24 for comparative studies.

**Table 5:** Quality assessment of the cohort studies considered, according to the Newcastle-Ottawa Scale (NOS).

Author	Selection *				Comparability **	Outcome ***			Score ****
	Representativeness of the exposed cohort ^1^	Selection of the non-exposed cohort ^2^	Ascertainment of exposure ^3^	Demonstration that outcome of interest was not present at start of study ^4^	Comparability of cohorts on the basis of the design or analysis ^5^	Assessment of outcome ^6^	Was follow-up long enough for outcomes to occur? ^7^	Adequacy of follow up of cohorts ^8^	
Fazal et al. [20]	c	c	a *	a *	a *	d	a *	a *	5
Donegan et al. [43]	a *	c	a *	a *	a *	b *	b	b *	6
Pedersen et al. [22]	a *	b	a *	a *	a *	b *	a *	a *	7
Dodd et al. [25]	b *	b	a *	a *	a *	b *	c	b *	6
Thomas et al. [44]	b *	c	a *	a *	a *	b *	a *	b *	7

* Maximum 1 point for each item. ** Maximum 2 points for each item. *** Maximum 1 point for each item. **** Maximum 9 points. 1: (a) Truly representative of the average _ (describe) in the community *; (b) Somewhat representative of the average __ in the community*; (c) Selected group of users e.g., nurses, volunteers; (d) No description of the origin of the cohort. 2: (a) Drawn from the same community as the exposed cohort *; (b) Drawn from a different source; c) No description of the origin of the non-exposed cohort. 3: (a) Secure record (e.g., surgical records) *; (b) Structured interview *; (c) Written self-report; (d) No description. 4: (a) Yes *; (b) No. 5: (a) Study controls for _ (select the most important factor) *; (b) Study controls for any additional factor * (These criteria could be modified to indicate specific control for a second important factor). 6: (a) Independent blind assessment *; (b) Record linkage *; (c) Self report; (d) No description. 7: (a) Yes (select an adequate follow up period for outcome of interest) *; (b) No. 8: (a) Complete follow up—all subjects accounted for *; (b) Subjects lost to follow up unlikely to introduce bias—small number lost - > ____% (select an adequate %) follow up, or description provided of those lost) *; (c) Follow up rate < ____% (select an adequate %) and no description of those lost; (d) No statement.

## Appendix A

### Appendix A.1. Searching Strategy

**Table A1:** Searching Strategy (Pubmed).

1	Rheumatoid Arthritis
2	Foot
3	Feet
4	Ankle
5	Bones of lower extremity
6	Hallux
7	First Metatarsophalang*
8	2 OR 3 OR 4 OR 5 OR 6 OR 7
9	1 AND (2 OR 3 OR 4 OR 5 OR 6 OR 7)
10	Surgic*
11	“Non-conservative treatment”
12	10 OR 11
13	9 AND 12
14	“Pain”
15	Disab*
16	Funct*
17	14 OR 15 OR 16
18	13 AND 17

### Appendix A.2. Scopus Search Strategy

((Rheumatoid Arthritis) AND (Foot OR Feet OR Ankle OR Bones of lower extremity OR Hallux OR First Metatarsophalang*) AND (Surgic* OR “non-conservative treatment”) AND (Pain OR Disab* OR Funct*))

### Appendix A.3. CINAHL Search Strategy

**Table A2:** CINAHL search strategy.

1	Rheumatoid Arthritis
2	Foot
3	Feet
4	Ankle
5	Bones of lower extremity
6	2 OR 3 OR 4 OR 5
7	1 AND (2 OR 3 OR 4 OR 5)
8	Surgic*
9	“Non-conservative treatment”
10	8 OR 9
11	7 AND 10
12	“Pain”
13	Disab*
14	Funct*
15	12 OR 13 OR 14
16	11 AND 15

### Appendix A.4. PROSPERO Search Strategy

((Rheumatoid Arthritis) AND (surgical) AND (foot OR ankle))
